# Understanding *Mycobacterium tuberculosis* complex in elephants through a One Health approach: a systematic review

**DOI:** 10.1186/s12917-022-03356-8

**Published:** 2022-07-06

**Authors:** Rajesh Man Rajbhandari, José de la Fuente, Dibesh Karmacharya, Sujala Mathema, Bijay Maharjan, Sameer Mani Dixit, Nisha Shrestha, João Queirós, Christian Gortázar, Paulo Célio Alves

**Affiliations:** 1 Dep. de Biologia, Faculdade de Ciências, BIOPOLIS, EBM & CIBIO-InBIO, Universidade do Porto; Institute for Game and Wildlife Research; Center for Molecular Dynamic-Nepal, Swaraj Sadhan, Thapathali 11, Kathmandu, Nepal; 2 SaBio. Instituto de Investigación en Recursos Cinegéticos, IREC (UCLM & CSIC), Ronda de Toledo s/n, 13005 Ciudad Real, Spain; 3 Department of Veterinary Pathobiology, Center for Veterinary Health Sciences, Oklahoma State University, Stillwater, OK 74078 USA; 4 Center for Molecular Dynamics Nepal, Kathmandu, Nepal; 5 Japan Nepal Tuberculosis Research Association, Kathmandu, Nepal; 6 CIBIO, Centro de Investigação em Biodiversidade e Recursos Genéticos, InBIO Laboratório Associado - BIOPOLIS Program in Genomics, Biodiversity and Land Planning. Campus de Vairão, Universidade do Porto, 4485-661 Vairão, Portugal; 7 Dep. de Biologia, Faculdade de Ciencias da Universidade do Porto, Rua do Campo Alegre, S/N, Edificio FC4, 4169-007 Porto, Portugal; 8 EBM, Estação Biológica de Mértola, Praça Luís de Camões, 7750-329 Mértola, Portugal

**Keywords:** *Elephas maximus*, *Loxodonta Africana*, *Mycobacterium tuberculosis*, Transmission, Prevention, Zoonosis

## Abstract

**Background:**

*Mycobacterium tuberculosis* complex (MTC) that causes the chronic infectious disease- tuberculosis (TB), often presents with a complicated epidemiological pattern where the transmission chain may include humans, domestic animals and wildlife, including elephants. TB has been reported globally in both captive and wild elephants. The One Health approach might be the most effective way of understanding the shared MTC infection dynamics in captive and wild animals like Asian elephants. This systematic review accumulates evidence on occurrence, transmission pathways, and preventive measures of TB in elephants from a One Health perspective.

**Results:**

The prevalence of TB reported in elephant populations ranges from 0 to 23.33% and high prevalence’s are reported for elephants that are in close proximity to infected humans. The risk of elephant to human infection transmission increased significantly with exposure duration and contact with infected elephants. Some studies described the plausible TB transmission to captive elephants from other animals (wild and domestic), suggesting inter- and intra-species transmission. The results of this systematic review based on 27 relevant published works, suggest three overarching interrelated transmission pathways for *M. tuberculosis* infections in Asian elephants- i) humans and elephants, ii) other animals (wild or domestic) and elephants and iii) unclear sources of infection.

**Conclusions:**

The progress made with new TB diagnostic tools provides multiple methods to choose from. However, lack of harmonization of TB testing in elephants and their human contacts remains a challenge to prevent TB in those animals. Routine TB screening among elephants and caretakers by setting up an occupational health program for early diagnosis of infection through combined efforts of public health, veterinary medicine, and occupational health experts is suggested. This implies the need for a One Health approach to elephant TB control. This review reveals the need for more research on *Mycobacterium tuberculosis* complex transmission pathways at the human-animal interface.

## Background

Tuberculosis (TB) affects humans, domestic animals, and wildlife populations [1, 2]. Among the members of the *Mycobacterium tuberculosis* complex (MTC) that cause TB in mammals, *M. tuberculosis* is the main causative agent of human and elephant TB. In addition, cases of *M. caprae* and *M. bovis*, also members of the MTC, were recently reported in a captive Asian elephant (*Elephas maximus*) and free-ranging African savanna elephants (*Loxodonta africana)*, respectively [3, 4].

Human cases of TB are reported almost in all countries of the world [5]. Generally, the human TB burden is high in the African and Asian nations, especially in low-income countries [6]. Amid the 20 high TB burden countries by the estimated absolute number of TB human cases, 14 countries (8 Asian and 6 African) are within the distribution range of wild elephants. The wild elephants are distributed in the African continent south of the Sahara, and south of the Himalayas throughout Southeast Asia and into China north to the Yangtze River [7]. The global distribution of wild or free-ranging elephants includes 27 countries, 13 in Asia and 14 in Africa [7]. Three elephant species are recognized, the Asian elephant, the African savanna elephant, and the African forest elephant (*Loxodonta cyclotis*) [8]. Among the Asian countries, India has the greatest number of elephants and the highest number of human TB cases [9]. Among the African nations, Botswana has the greatest number of wild elephants and South Africa has the highest number of human TB cases [10]. Countries that have a high prevalence of TB in humans such as Nepal and Thailand also have TB in elephants [11, 12]. There are also around 15,000 to 20,000 captive elephants spread worldwide in zoos, circuses and others private owners [13].

TB in elephants was recognized more than 2000 years ago [14]. The first case of TB in elephants was reported in London Zoo, 1875 [15]. Sporadic cases of TB in captive Asian elephants were reported in the early twentieth century. It was only in the mid-twentieth century, that the first case in an African elephant was reported. After the initiation of systematic surveillance in 1998 in the U.S, the number of TB cases in elephants is rising [6]. However, there is a lack of a robust surveillance system to report TB cases and deaths linked to TB in elephants. The main causative agent for TB in elephants is *M. tuberculosis* [5]. Captive elephants are more prone to TB as they are often in close and frequent contact with potentially infected human beings. It is presumed that there are possibilities of *M. tuberculosis* transmission between humans and elephants or between wild and domestic elephants [16]. The direction of transmission (elephant to elephant, elephant to human or human to elephant) has not been determined in most cases. Likewise, it has been suggested that cattle with close contact with humans could get infected with *M. tuberculosis* and eventually transmit it to elephants via contaminating the same grazing field as used by elephants [16]. However, to date there is no documented transmission of *M. tuberculosis* between elephants and livestock. There are limited reports on TB in free-ranging elephants [9, 16–19].

Given that TB is present in both captive [1, 2, 20, 21] and free-ranging wildlife [2], it represents a considerable zoonotic risk. Some evidence suggests infection of *M. tuberculosis* in a variety of domestic animals such as dogs [22] and cattle [23], as well as in non-domestic animal species including elephants [24–27]. In some species such as cattle and goats [28], *M. tuberculosis* infections are self-limiting and persistence of the pathogen in the population does not occur without repeated exposure to human cases [29]. However, the dynamics of *M. tuberculosis* transmission among wild animal species remains uncertain [30] as no evidence of *M. tuberculosis* infection in wildlife outside zoos could be observed [31]. A holistic understanding is needed on transmission pathways of zoonosis and reverse zoonosis of *M. tuberculosis* and other members of *MTC* in different species, especially in elephants. Thus, the present study aims to accumulate the evidence on the occurrence, transmission pathways, and preventive measures of TB in elephants.

## Results

### Study selection and study characteristics

From the 122 articles selected for the abstract review 54 articles passed the filters. After the full-text reviews, 27 articles were selected for the study with focus on studies on prevalence of TB in elephants, dynamics of MTC transmission, and preventive measures of TB. A total of 27 articles described the possible chain of transmission and major preventive measures. Among those articles, there were 14 epidemiological, one clinical research, four outbreak investigations and two review articles. A total of 14 studies assessed the prevalence of TB in elephants, 12 being epidemiological studies and one clinical research. None of the articles with outbreak investigations and review articles had sufficient evidence to determine the prevalence of TB in elephants.

### Main findings: assessment and prevalence

The seroprevalence of TB in elephants varied from 0 to 23.33%, however, there is a variation of seroprevalence between wild and captive elephants, African and Asian elephants. The TB seroprevalence among captive Asian elephant’s ranges from 15.2 to 23.33% [32–36]), while in captive African elephants is approximately 17% [10]). The point prevalence of *M. tuberculosis* infection in Asian elephants was 5.1% for the time period of 1997 to 2011, while it remained 0 in African Elephants for the same time period [37]. There are also sporadic reported TB cases in wild Asian elephants as well as wild African elephants. However, TB prevalence in wild elephants has not been adequately studied to fully understand its dynamics and transmission pathways. The evidence indicates wild elephants can maintain human TB in the wild and that the infection can be fatal [9, 17].

We identified increased risk of TB in elephants with the growth in exposure to potentially TB infected humans or animals including other elephants, and vice versa. The studies used a wide variety of diagnostic measures throughout the years. Culture of the trunk-wash sample was preferred as a diagnostic tool before more rapid methods of TB diagnosis became available. We found the application of more than one diagnostic method to determine the prevalence of *M. tuberculosis* infection in elephants, which signals the advancements made over the years in the diagnostic technologies. The gradual innovation of TB diagnostic tools has provided multiple available methods for diagnosis. For instance, elephant TB Stat-Pak has been replaced by Dual Path Platform (DPP) TB test [38] (Tables 1 and 2).

**Table 1:** Main findings and seroprevalence and *M. tuberculosis* infection prevalence of included studies using serology and culture methods, respectively

S.N	Authors	Research Design	Type of diagnosis	Diagnostic method	Country	Elephant species	Source	Prevalence (%)	Main findings
1	Mikota et al. (2000) [15]	Cross sectional study	Culture	Culture	USA	Asian Elephant	Captive and Free-ranging	3.30%	Further research is essential to validate other diagnostic tests and treatment protocols.
2	Abraham (2009) [32]	Cross-sectional study	Serology	Elephant TB Stat-Pak	India	Asian Elephants	Captive	15.20%	The rapid serum test showed that not less than 15.2% of the population has seropositivity to *M.bovis*
3	Murphree et al. (2011) [20]	Cohort study and onsite assessment	Culture	Culture of environmental samples and trunk wash.	USA	Asian and African elephant	Refuge	All were Negative except for specimens taken in December 2008 from 1 elephant living in the quarantine area	The risk for conversion was increased for elephant caregivers and administrative employees working in the barn housing
4	Verma et al. (2012) [33]	Cross-sectional study design	Culture & Serology	Culture, ELISA, Immunoblot analysis	India	Asian elephant	Captive	15.90%	High prevalence of asymptomatic *M. tuberculosis* infection in Asian elephants in a captive Indian setting.
5	Feldman et al. (2013) [37]	Prevalence study	Culture	Culture of trunk wash sample (n1 = 684 Asian, and n2 = 459 African)	USA	Asian elephant	Captive	5.10%	The incidence of tuberculosis differed significantly between Asian and African elephants. Accurate and species-specific knowledge of prevalence and incidence will inform our efforts to mitigate occupational risks associated with captive elephants in the USA.
6	Ong et al. (2013) [36]	A cross-sectional study	Serology	Elephant TB Stat-Pak assay/ TB antigen rapid test, trunk wash samples, QuantiFERON-TB Gold ELISA	Malaysia	Asian elephant	Captive	20.40%	There was evidence of active and latent TB in the elephants and the high seroprevalence in the elephants and their handlers suggests frequent, close contact, two-way transmission between animals and humans within confined workplaces.
7	Yakubu (2015) [34]	Cross sectional study	Molecular	PCR	Malaysia	Asian elephant	Captive	23.33%	Risk of infection to be significantly associated with facility staff, workers older than 30 years of age, mahouts, and foreigners.
8	Mikota et al. (2015) [39]	Prospective study	Serology & Culture	Elephant TB Stat-Pak assay, Culture	Nepal	Asian Elephant	Captive	0%	Culture and serological results were variable and required careful interpretation to develop criteria to assess TB risk.
9	Yakubu et al. (2016) [40]	Cohort and Cross-sectional study	Serology	STAT-PAK and DPP VetTB Assays, QuantiFERON-TB Gold In-tube test (QFT) were used.	Malaysia	Asian elephant	Captive	23.30%	Univariate analysis showed that elephants with assigned mahouts have significantly higher risk of TB OR = 3.8 The risk of seroconversion was significantly higher among elephants with assigned mahouts [OR = 4.9]
10	Magnuson et al. (2017) [41]	Clinical research	Culture and Molecular	Culture, PCR	USA	Asian elephant	Captive	8.40%	Molecular test results can be used to support current diagnostic procedures applied by veterinarians for treatment decisions to prevent the spread of tuberculosis in elephants.
11	Rosen et al. (2018) [10]	Cross sectional study	Serology	Elephant TB Stat-Pak and DPP VetTB Assay	Zimbabwe	African elephant	Captive	17.10%	Strong correlations were noted between contact with wild elephants and facilities (*p* = 0.86)
12	Jeewan et al. (2021) [35]	Cross sectional study	Serology	Elephant TB Stat-Pak	Nepal	Asian elephant	Captive	21.56%	The occurrence of TB seropositive cases in other more remote national parks suggest TB may be wide spread among the captive elephant population of Nepal

*PCR* Polymerase chain reaction, *ELISA* Enzyme-linked immunosorbent assay, *TB* Tuberculosis

**Table 2:** Main findings and seroprevalence and *M. tuberculosis* infection prevalence of included studies using other tests

S.N	Authors	Research Design	Diagnostic methods	Elephant species	Source	Country	Prevalence (%)	Main findings
1	Larsen et al. (2000) [42]	Cross-sectional observational study design	ELISA for Screening	Asian and African elephant	Captive	USA	14.89%	Multiple-antigen ELISA would be a valuable screening test for detecting *M. tuberculosis* infection in elephant herds.
2	Zachariah et al. (2017) [16]	Surveillance	PCR, postmortem examination	Asian elephant	Free-ranging	India	3.40%	Exposure of bulls to humans infected with TB during conflict activities could be the possible explanation regarding the transmission of disease. All 3 animals were emaciated and considered TB to be the cause of death.

### Transmission pathways and preventive measures

We analyzed a total of 27 studies to assess the transmission pathways and preventive measures. The studies were carried out in diverse elephant populations including captive and wild Asian and African elephants. We identified more studies among captive elephants than among wild ones. These studies were conducted across different continents - America, Africa, Asia, and Europe. Three overarching interrelated themes for transmission pathways were identified: between humans and elephants, other animals to elephants, and unclear source of infection. Several studies suggested transmission between humans and elephants [1, 17, 20, 21, 31, 36, 43, 44]. Few of the studies described the plausible transmission from other animals (wild and domestic) to elephants [5, 43–45]. Several studies lacked information on the source of infection [9, 11, 18].

Studies of TB in captive elephants from Thailand, Malaysia, Switzerland, USA, Nepal, India, and some African countries revealed possible transmission of *M. tuberculosis* between elephants and humans [1, 10, 12, 20, 34, 38]. One of the studies carried out in Southern India among wild elephants indicated that TB might be spilling over from humans (reverse zoonosis) and emerging in wild elephants [16]. Similarly, evidence from Kenya and India showed wild elephants could harbor *M. tuberculosis* and revealed that the infection could be fatal. However, the transmission sources of infection were unclear regarding if the infections originated from humans or other animals [9, 18]. Likewise, studies in Sweden, Australia, and the U.S. indicated possible transmission from elephants to elephants as well as to other captive animals, especially in zoos [5, 21, 44]. Hence, it signifies the need for a One Health approach to combat TB due to probable transmission pathways between humans, elephants, and other animals. These pathways could be partly mediated by environmental matrices such as shared water or feed [46].

We identified possible preventive measures for *M. tuberculosis* transmission and infection control measures. The possible chain of transmission could be interrupted through routine TB screening among elephant handlers, occupational health programs, early diagnosis and treatment of infections among the elephant handlers, isolation of infected elephants and improving TB screening methods for elephants especially among the captive elephants [47, 48]. Additionally, it is essential to perform screening of newly acquired elephants, isolation of infected elephants and early treatment of confirmed cases in captive elephants. Likewise, it is important to ensure TB screening of captive elephants before releasing them into the wild [16, 18]. Similarly, the exposure could be reduced by minimizing shared feed with other wildlife. For early detection and efficient treatment, accurate antibody tests such as the DPP Vet TB test are already available [36]. However, better ways to identify culture-positive elephants are still needed given the limitations of trunk wash. Thus, there is a need to develop a blood antigen test that can identify culture-positive elephants with improved sensitivity [49]. One measure regarding diagnosis in elephants is to use a combination of diagnostic approaches as single diagnostic measures cannot always identify TB [34, 36] (Table 3).

**Table 3:** *Mycobacterium tuberculosis* transmission pathways and preventive measures

S.N	Study references	Elephant species	Source	Country	Disease transmission	Preventive measures
1	Zachariah et al. (2017) [16]	Asian elephant	Free-ranging	India	Between human and elephant	Continued Surveillance among elephant population.
2	Obanda et al. (2013) [18]	African elephant	Free-ranging	Kenya	Source of infection is unclear	Domesticated elephants into the wild require efficient screening for TB, the status of TB in wild elephants should be assessed.
3	Angkawanish et al. (2010) [12]	Asian elephant	Captive	Thailand	Between human and elephant	Early diagnosis of infection is necessary. Combination of diagnostic approaches is essential.
4	Yakubu (2015) [34]	Asian elephant	Captive	Malaysia	Between human and elephant	The need for control strategies such as screening and newly acquired elephants, isolation of infected elephants and early treatment of confirmed cases.
5	Ong et al. (2013) [36]	Asian elephant	Captive	Malaysia	Between human and elephant	Elephant handlers need to be aware of the risk of TB acquisition from infected animals and be educated concerning infection control measures.
6	Murphree et al. (2011) [20]	Asian and African elephant	Refuge	USA	Between human and elephant	Increased knowledge about MTB infection in elephants, improved infection control practices, and specific occupational health programs.
7	Feldman et al. (2013) [37]	Asian and African elephant	Captive	USA	The difference in species susceptibility	Mandatory annual tuberculosis screening for all the elephants.
8	Rosen et al. (2018) [10]	African elephant	Captive	Zimbabwe	Source of infection is unclear	Routine TB testing of elephant handlers and regular serological screening of elephants are recommended as preventive measures.
9	Michalak et al. (1998) [1]	Asian and African elephants	Free-ranging	USA	Between human and elephant	Veterinary practices should be initiated to reduce the risks of exposure to animals infected with MTB.
10	Chandranaik et al. (2017) [9]	Asian elephant	Free-ranging	India	Source of infection is unclear	Wild elephants can harbor MTB that can become fatal. Need to assess the status of TB among wild animals and to examine whether wildlife can be a potential reservoir of the disease.
11	Ghielmetti et al. (2017) [30]	Asian elephant	Captive	Switzerland	Between human and elephant	Different transmission chains or prolonged infection over time.
12	Magnuson et al. (2017) [41]	Asian elephant	Captive	USA	Between human and elephant	Molecular test results can be used to support current diagnostic procedures applied by veterinarians for treatment decisions to prevent the spread of tuberculosis in elephants.
13	Rosen et al. (2018) [10]	African elephant	Captive	Five countries of Africa	Between human and elephant	Minimizing exposure through shared feed with other wildlife, routine TB testing of elephant handlers, and regular serological screening of elephants are recommended as preventive measures.
14	Simpson et al. (2017) [50]	Asian and African elephant	Captive	USA	Between human and elephant	Infection control protocols and careful monitoring of the treatment of captive elephants with tuberculosis are warranted.
15	Yakubu et al. (2016) [40]	Asian elephant	Captive	Malaysia	Between human and elephant	The need for TB screening of newly acquired elephants, isolating seropositive elephants and performing further diagnostic tests to determine their infection status, and screening elephant handlers for TB, pre- and post-employment.
16	Zlot et al. (2016) [21]	Asian elephant	Captive	USA	Between human and elephant	Improved TB screening methods for elephants are needed to prevent exposure of human contacts.
17	Lassausaie et al. (2015) [43]	Asian elephant	Captive	Laos	Between human and elephant	Medical monitoring of people working or living with elephants should thus be implemented.
18	Paudel et al. (2014) [11]	Asian elephant	Captive	Nepal	Between human and elephant	Regular TB screening of elephant handlers to safeguard human health and help prevent transmission of TB from humans to elephants.
19	Lewerin et al. (2005) [5]	Asian elephant	Captive	Sweden	Between Elephants and Captive Animals	Elephants and giraffe were found to have been infected by four different strains of MTB in a large Swedish zoo.
20	Larsen et al. (2000) [42]	Asian and African elephant	Captive	USA	TB infected elephants are the potential source of infection	Multiple-antigen ELISA would be a valuable screening test for detecting MTB infection in elephant herds.
21	Paudel et al. (2018) [38]	Asian elephant	Captive	Nepal	Source of infection is unclear	Regular TB screening of elephant handlers to safeguard human health and help prevent transmission of TB from humans to elephants.
22	Stephens et al. (2013) [44]	Asian elephant	Captive	Australia	Elephant to Humans and other animals	The mechanism for transmission from elephants in the Australian zoo require further investigation.
23	Montali et al. (2001) [2]	Asian Elephant	Captive and Free-ranging	USA	Transmissions of MTB between animals and humans are uncommon	Programs for tuberculosis prevention in animal handlers.
24	Mikota & Maslow (2011) [51]	Asian Elephant	Captive	USA	Between human and elephant	More epidemiological investigation in the transmission.
25	Greenwald et al. (2009) [45]	Asian and African elephant	Captive	Europe and the USA	Elephant to Humans and other animals	Rapid and accurate antibody tests to identify infected elephants will likely allow earlier and more efficient treatment, thus limiting the transmission of infection to other susceptible animals and to humans.
26	Mikota et al. (2000) [15]	Asian Elephant	Captive and Free-ranging	USA	Elephant and human	Further research is essential to validate other diagnostic test and treatment protocols
27	Jeewan et al.(2021) [35]	Asian elephant	Captive	Nepal	Between elephant to other hosts, including humans	Include blood parameters in future TB surveillance studies.

*MTB M. tuberculosis*, *ELISA* enzyme-linked immunosorbent assay, *TB* tuberculosis

## Discussion

This review revealed that TB in elephants is widespread across the globe. In general, the rates of *M. tuberculosis* infection are higher in Asian elephants compared to African elephants [52]. Confirmed *M. tuberculosis* infections are reported both in wild and captive elephants across different countries, although being more frequent in captive elephants. The same *M. tuberculosis* strain in an elephant and a handler has been reported in only one case [1]. However, most human cases have been diagnosed by tests such as the intradermal tuberculin test, Quantiferon, chest Xray, among others, which do not identify the mycobacteria strain involved [53–57]. Therefore, multidisciplinary interventions with intersectoral coordination must be implemented to combat TB in elephants and humans.

Even though the cases of direct *M. tuberculosis* transmission are apparently rare, there are odds of transmission when infected elephants are at an advanced stage of this disease. Due to paucity of research in wild elephants, the occurrence of *M. tuberculosis* was mostly observed in captive elephants. For instance, *M. tuberculosis* isolates were extracted from two elephants of Chitwan National Park and one elephant of Koshi Tappu Wildlife Reserve of Nepal. These elephants were in contact with domestic and wild animals like rhinos and different deer species along with their handlers [11]. Additionally, in a serosurveillance program of captive elephants in Nepal, out of 153 elephants, 21.56% were serologically tested positive [35]. There are evidences that human handlers might act as a source of infection for the animals [58]. Likewise, at a zoo in the US, *M. tuberculosis* was diagnosed in an elephant, a rhino and three mountain goats [31]. Seven out of 24 keepers in the south-east zoo in the U.S. tested positive in intradermal tuberculin tests and were assumed to be infected by airborne transmission from an affected white rhinoceros. In this regard, a white rhinoceros tested positive for *M. bovis* that later spread to colobus monkeys [59]. In a separate study, *M. tuberculosis* strains isolated from captive elephants in Thailand seemed to have originated from humans [12]. The transmission of *M. tuberculosis* from elephant-to-elephant and elephant to other animals is also possible [1, 4, 48, 59]. Yet, a clear transmission pathway and the source of *M. tuberculosis* infection between animals has not been established [44, 47, 60].

Many countries are dependent on elephant-related industries such as agriculture and tourism leading to more interaction between humans and elephants [17]. The tourism activities lead to increased interaction between captive elephants, humans and wild elephants. TB in wild elephants is an emerging challenge. There are few studies reporting TB among wild elephants [8, 16, 17, 54]. Still, more investigation is needed to promote further epidemiological studies among wild elephants [48]. The surveillance and epidemiological studies on wild elephants are more complicated as it is difficult to track the exact location, number, and duration of potential exposure [17]. There are multiple diagnostic and screening tools available to assist in the diagnosis of MTC in elephants. Even so, confirmation of the true diagnosis of the clinical disease remains challenging [60, 61].

In the absence of highly sensitive diagnostic assays; a combination of routine medical examination could be recommended [60]. Standard tests such as VetMAX™ MTBC qPCR Kit [62], and serological tests using Elephant TB STAT-PAK,® DPP VetTB® Assay, MAPIA (multi-antigen print immunoassay) [38], and interferon gamma release assay (IGRA) [49] are used for detection of MTC in different parts of the world based on accessibility.

Often, TB is transmitted from droplet nuclei (i.e., respiratory secretions). *M. tuberculosis* has been isolated from respiratory secretions, trunk washes, faeces, and vaginal discharges in elephants [63]. TB can also be transmitted from elephants to humans [20, 21]. Transmission of elephant *M. tuberculosis* to humans is more likely an occupational health concern rather than a general public health concern [1, 20, 48]. However, with the increased use of elephants in the entertainment and tourism sector, it is likely to increase *M. tuberculosis* transmission beyond the occupational health concern.

Therefore, the One Health perspective consisting of activities like adopting infection control measures in the captive environment, routine TB screening among elephant handlers, occupation health programs, and establishing a mechanism for early diagnosis of infection among elephants is recommended. Additionally, it is essential to ensure screening of TB before the release of captive elephants into the wild as well as before adapting wild elephants into the captive environment [18]. Furthermore, elephants with active TB should be segregated and treated, which will aid in the prevention of *M. tuberculosis* transmission between species and will contribute to the conservation of elephants [48]. Although caution is needed considering antibiotic resistance.

The review is limited to the systematic analysis of the literature and has not executed a meta-analysis. The findings of the study are based on a relatively limited number of studies; thus it is challenging to fit into the PRISMA format. There is a high variation in study design, sample size, screening, and diagnostic tools used in the included studies. Thus, it is difficult to compare the best method because of the variety of diagnostic tests employed. Nevertheless, this review highlights the need to carry out more epidemiological research in both wild and captive elephant populations to determine the exact prevalence, chain of transmission, and other related factors. There is a huge gap in the evidence on the dynamics of *M. tuberculosis* transmission between other animals and elephants, reinforcing the need to promote research to develop innovative and robust screening and diagnostic tools for the early diagnosis of *M. tuberculosis* and other members of the *M. tuberculosis* complex in elephants. This would help to reduce the risk of TB in elephants as well as in the in-contact human population and further contribute to prevent possible transmission to other animal populations.

## Conclusion

*M. tuberculosis* infection has affected the elephant population, and in particular, Asian elephants. Recent studies suggest human-elephant and elephant-human *M. tuberculosis* transmission. It is a public health concern that needs a One Health perspective with a combined effort of biologists, public health and occupational health experts, and veterinarians to reduce the occurrence of zoonosis and reverse zoonosis of *M. tuberculosis*.

## Methods

### Overview

This is a systematic review grounded on the preferred reporting items for systematic reviews and meta-analyses (PRISMA) guidelines [64]. The keywords like *Mycobacterium tuberculosis*, prevalence, transmission, prevention, and elephants were scanned in Google Scholar, PubMed, Science Direct, and Web of Science. The articles were explored without the time constraints and the articles were sorted for relevance with the keywords.

### Literature search

**Fig. 1:**
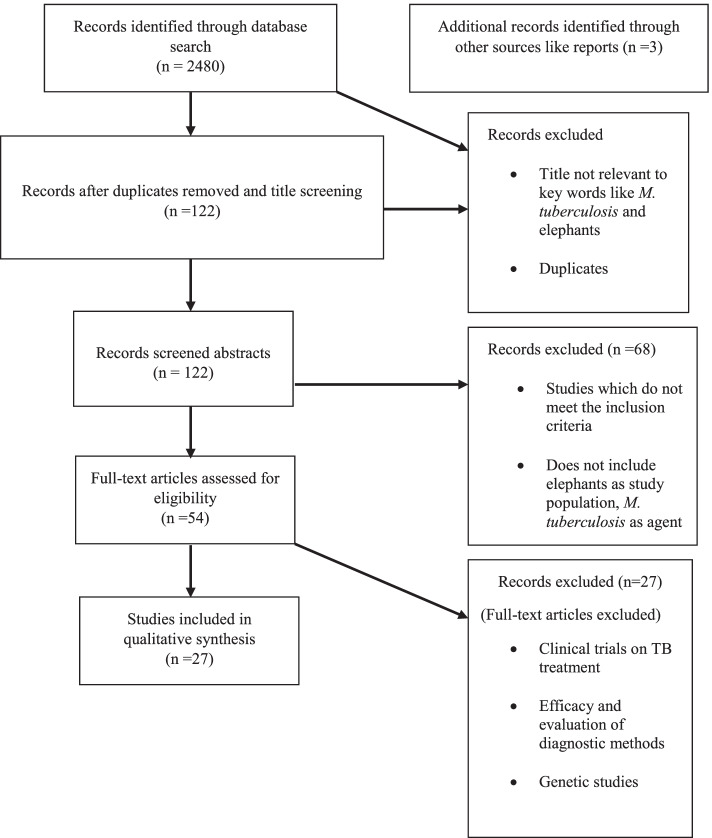
Flowchart of the systematic review

A total of 2480 articles were obtained during the first search. Then, the identified articles were saved in Zotero (a reference management software) [65]. Duplicate articles were sought using the title, DOI, and ISBN fields in Zotero. If these fields match (or are absent), years of publication (if they are within a year of each other) and author/creator lists (if at least one author last name plus first initial matches) were explored to determine the replicas. The identical articles were managed by merging them, rather than deleting one of the duplicates. Furthermore, the titles of the articles were assessed and the articles that were not relevant to keywords were excluded. A total of 122 articles were selected for the abstract review. A report of all the articles with titles and abstracts were generated in a file. The abstracts of the articles were reviewed, and 54 articles were selected for full-text review based on various parameters (Table 4). After the full-text review, 27 articles were selected for the study, focusing on studies about the prevalence of TB in elephants, dynamics of *M. tuberculosis* transmission, and preventive measures of TB. The selected articles were entered in MS-excel version 13.0 including the major outcomes of the studies. Then, the findings and major outcomes were analyzed and interpreted for the result of the study (Fig. 1). Furthermore, additional literature like reports on the global distribution of elephants, country-wise elephant’s population, distribution of TB in humans, and high TB burden countries were also reviewed.

**Table 4:** Inclusion and exclusion criteria peer-reviewed literature

Parameter	Inclusion	Exclusion
Study design/ type	• Meta-analysis or systematic review	• Narrative review
	• Randomized controlled trails	• Non-pertinent publication types (e.g., expert opinions, letters
	• Non-randomized, prospective comparative studies	• Editorials, comments, conference,
	• Prospective observational studies (e.g., cohort studies)	• Abstract/poster, news, consensus document, chapter
	• Retrospective observational studies (e.g., case-control studies)	
	• Cross-sectional studies	
	• Case studies	
	• Outbreak Investigation	
	• Clinical studies	
	• Short communication	
Study Quality	• Number of subjects (no minimum)	• Insufficient methodological quality (both inherent methodology as well as an insufficient description
	• Study duration (no minimum)	
Study Population	• Wild and captive elephants	• Other animals (wild and domestic animals) which are not the contacts of elephants
	• Contacts of wild and captive elephants (Human and other animals)

### Article inclusion and exclusion criteria

The inclusion and exclusion criteria were established on different parameters like study design/type, quality of the study, and the study population. The studies like meta-analysis/systematic review, randomized controlled trials (RCTs), prospective studies, cohort studies, cross-sectional studies, case studies, outbreak investigation, and clinical studies were involved in the review. On the other hand, narrative reviews, non-pertinent publications, opinions, news articles, abstracts were omitted in the review. Similarly, studies with any time duration or number of the study population were also embraced for the review. Contrarily, research with insufficient methodological quality as well as the insufficient description was barred from the review. Likewise, research carried out in wild and captive elephants including their contacts were encompassed in the review. Studies on TB in other animals were excluded from the review. The details of the inclusion and exclusion criteria are listed in Table 4.

### Search strategy with databases

The search was performed until 2021 and included only the articles that were published in English. The newspaper articles, blog posts, conference abstracts, narrative reports, editorials, and field visit reports were excluded for the study.

### Data extraction

Two authors screened the search results. The following information was extracted from each paper: publication year, country, type of elephants, species of elephants, study design, sample size, the sample used for diagnosis, diagnosis methods, prevalence, mortality, and major outcome. The affiliation of authors, methods of diagnostic tools used, and type of elephant included in the study, and investigated research methods were noted to sort out the research. After the full article review, based on the above-mentioned information, authors reached the consensus to include the article for review.

### Risk of Bias and article quality

The sources of bias were assessed for the articles that met the inclusion criteria. The studies with screening and diagnostic tests for TB in elephants were included in the review. So, the common sources of bias in diagnostic accuracies like partial verification bias, clinical review bias, and observer or instrument variation bias were assessed in the articles. The authors ensured the methodological issues including content and methods, which included consistency assessment of 27 studies between the authors. Besides, studies were assessed for a sufficient description of the methodology used. The studies with an insufficient description of the methods were not included in the study. Furthermore, the authors re-checked for the missing information such as study population, design, and outcome of the research.

## Data Availability

The datasets used and/or analyzed during the current study are available from the corresponding author on reasonable request.

## Abbreviations

*M. tuberculosis*: *Mycobacterium tuberculosis*
MTC: *Mycobacterium tuberculosis complex*
PRISMA: Preferred reporting items for systematic reviews and meta-analyses
ELISA: Enzyme-linked immunosorbent assay
MAPIA: Multiantigen print immunoassay
DPP: Dual path platform.
qPCR: Quantitative real-time Polymerase chain reaction
IGRA: Interferon gamma release assay
